# Racial Bias Correlates with States Having Fewer Health Professional Shortage Areas and Fewer Federally Qualified Community Health Center Sites

**DOI:** 10.1007/s40615-021-01223-0

**Published:** 2022-01-10

**Authors:** Lonnie R. Snowden, Eli Michaels

**Affiliations:** 1 Health Policy and Management Division, School of Public Health, University of California, Berkeley, CA USA; 2 Epidemiology Division, School of Public Health, University of California, Berkeley, CA USA

**Keywords:** African Americans, Racial bias, Healthcare disparities, Poverty policy, Federally Qualified Health Centers

## Abstract

Federally Qualified Community Health Centers (FQHCs), serving Health Professional Shortage Areas (HPSAs), are fixtures of the healthcare safety net and are central to healthcare delivery for African Americans and other marginalized Americans. Anti-African American bias, tied to anti- “welfare” sentiment and to a belief in African Americans’ supposed safety net dependency, can suppress states’ willingness to identify HPSAs and to apply for and operate FQHCs. Drawing on data from *n* = 1,084,553 non-Hispanic White Project Implicit respondents from 2013–2018, we investigated associations between state-level implicit and explicit racial bias and availability of FQHCs and with HPSA designations. After controlling for states’ sociopolitical conservatism, wealth, health status, and acceptance of the Affordable Care Act’s Medicaid expansion, greater racial bias was correlated with fewer FQHC delivery sites and fewer HPSA designations. White’s bias against African Americans is associated with fewer FQHC opportunities for care and fewer identifications of treatment need for African Americans and other low-income people lacking healthcare options, reflecting bias-influenced neglect.

Federally Qualified Health Centers (FQHCs), federally funded programs offering primary and preventive healthcare from thousands of sites nationwide [1], are key but overlooked components of the US safety net. FQHCs grew by 211% from 2000 to 2019 and served almost 30 million people [2]. FQHCs target Health Resources and Services Administration–identified Health Professional Shortage Areas (HPSAs) which certify limited healthcare access in Medically Underserved Areas (MUAs), Medically Underserved Populations (MUPs), and Medically Underserved Institutions (MUIs).

Many Americans disapprove of safety net programs such as FQHCs, condemning them as “welfare” for the “undeserving poor” [3, 4]. African Americans are closely identified with “welfare”: research participants depict prototypical “welfare” recipients as African American [5] and most Americans mistakenly think that African American “welfare” recipients are equal to or greater in number than White “welfare” recipients [6]. Welfare recipient and African American stereotypes are now so intertwined that political scientists propose that “racial resentment” [7], a belief that African Americans lack values of self-reliance, work ethic, and discipline [8] and are disposed to “welfare” dependency, is the principle expression of politically consequential contemporary racism [9].

Racially biased, anti- “welfare” sentiments limit access and generosity of safety net benefits for everyone in need [10]. Skeptical states choose restrictive approaches when implementing Temporary Assistance for Needy Families (TANF) [11], Supplemental Assistance for Needy Families (SNAP, or food stamps) [12, 13], unemployment insurance [14], and Medicaid [15, 16], and they refused Medicaid expansion [17]. Reservations about safety net programs can extend to FQHCs and HPSAs. States with fewer FQHC sites have lower TANF participation rates and ranked lower in preparedness for Medicaid expansion; states with fewer designated HPSAs paid lower SNAP per person benefits and had fewer non-elderly adults on their SNAP caseloads [18].

Wider safety net restrictions reflect political and cultural disapproval of safety net programs and “welfare” recipients, and they can limit state governments’ willingness to assist impoverished citizens [19, 20]. During the presidency of President George W. Bush and “compassionate conservatism” and sometimes later [21], FQHCs expanded with wide political support [22]. More recent FQHC growth was stimulated by President Obama’s American Recovery and Reinvestment Act [21], and especially by the polarizing Affordable Care Act (ACA) [23] which featured Medicaid expansion prominently [9, 24, 25]. The ACA is strongly associated in the public mind with the African American President Obama (“Obamacare”) [9, 10], arousing disapproval generalizing to government involvement in healthcare beyond the ACA [9]. FQHCs are increasingly swept up in political polarization: President Biden’s American Rescue Plan, which expanded FQHC funding and included other safety net program increases, passed with no Republican votes [26]. A disapproving public and state elected officials and administrators can discourage some state officials from recruiting and supporting applications for FQHCs and identifying HPSAs.

Collective racial biases, those shared by county or state residents, are gaining recognition as a social determinant of health. Evidence and theory suggest that studying collective bias—that is, bias aggregated to area-levels—yields stronger and more stable effects than studying bias at the individual-level [27–29]. Various data sources capture regional rates of anti-racial, ethnic, or immigrant group sentiments and beliefs, which can be linked to inequities in health and social outcomes [30]. A prominent example is Project Implicit, a multiyear national database containing more than 1.5 million assessments of implicit and explicit racial bias [31]. Explicit bias refers to conscious and overt attitudes, whereas implicit racial bias is unconscious and hard-to-control associations: for example, of African Americans with negative characteristics and Whites with positive characteristics. Researchers have linked county-level rates of pro-White/anti-Black racial bias [32] with racial inequities in infant health [33], COVID-19 illness [34], and death from circulatory diseases [35].

State-level bias has been associated with Medicaid generosity [15], and circumstantial evidence associates state-level bias with states having fewer FQHCs. In 10 states with the highest proportions of African Americans—including Deep South states with relatively unhealthy citizens [36] and demonstrating high levels of racial resentment [20]—FQHCs grew at two-thirds the rate as in states where African Americans’ representation is less [37] (calculated from Fig. 2 data in ref. 37). However, direct evidence currently is lacking associating states’ racial bias itself with having fewer FQHCs.

The prospect of HPSA certification and prospective FQHC applications can trigger anti-African American, anti- “welfare” bias for several reasons. Federal HPSA designations evoke federal commitment to aid the poor, and they include heavily African American Medically Underserved Populations and Medically Underserved Communities. HPSAs sometimes target public housing and homeless populations where African Americans are overrepresented. FQHCs receive direct federal support and rely heavily on public insurance, principally Medicaid, for financing. FQHC importance for African Americans’ healthcare was recognized by the Congressional Black Caucus (CBC): among its funding priorities for COVID-19 relief, the CBC requested greatly increased funding for FQHCs [38].

The present study investigated whether White state residents’ implicit and explicit pro-White/anti-Black biases toward African Americans are associated with states having fewer FQHCS and HPSAs. States are important units of analysis because, under “federalism” [19], states insist on, and in fact play, key roles in implementing safety net policy [12–14, 19, 25], and because states vary in anti- “welfare”, anti-Black sentiments [15, 18]. Only one previous study examined state-level racial bias’s impact on safety net programming. Using data from Project Implicit, Leitner et al. [15] found that states higher in anti-Black racial bias spent less on Medicaid payments to disabled persons. 

Because bias-influenced, anti- “welfare” sociopolitical cultures may restrain state officials considering supporting FQHC applicants and operations and hinder officials developing cases for HPSA designation, we assessed whether states where White Project Implicit respondents showing higher levels of implicit and explicit anti-Black/pro-White racial bias levels had fewer areas designated as HPSAs and fewer FQHC sites. We focused on HPSA designations because they are state-mediated representations of federally recognized healthcare inaccessibility and because they make possible federal involvement for addressing limited healthcare resources for African Americans and other poor state residents. We focused on FQHCs because successful FQHC applicants and programs often receive state-level support [21] and because many centers operate multiple sites. In wider perspective, our study advances bias theorist proposal [28] that, by pursuing bias-influenced policy more than individual’s biases, we might better illuminate pathways for overcoming bias’s deleterious effects.

## Methods

We examined associations between states’ implicit and explicit bias levels and availability of FQHCs and HPSA designations for poor adult state residents. We assessed FQHC sites and HPSA designations per-poor non-elderly adult because this population is stigmatized as “underserving poor” and is a target of race-infused anti-welfare sentiments [3, 5, 10] and because elderly persons 65 and over and children, who are considered “deserving poor,” have publicly sponsored coverage and healthcare options through Medicare and the Children’s Health Insurance Program, respectively.

Following others [35, 39], we examined the racial bias of non-Hispanic Whites only because this group has the most wealth and decision-making power in the USA and most likely harbors bias-related “racial resentment” of “welfare” [40], and because Black’s anti-Black bias measures a phenomenon sometimes interpreted as “internalized racism” [41] and not paralleling White’s anti-Black bias. When studying FQHCs, we controlled for conservatism of states’ residents, as well as income, health status, and whether official elected ACA’s expanded Medicaid given the opportunity in 2014. When studying HPSAs, we controlled for the variables identified above and, because FQHCs are HPSAs, we controlled for FQHCs.

### Dependent Variables

We investigated State’s Federally Qualified Health Center Sites and Health Professional Shortage Areas per non-elderly poor resident. FQHCs are a small subset of HPSAs and accordingly the two are correlated. We adjusted for this interdependency in our analysis, as described below.

#### State’s Federally Qualified Health Center Sites per Non-elderly Poor Resident

FQHCs meet federal health center requirements and receive federal grants under Sect. 330 of the Public Health Service Act. Kaiser Family Foundation compiles and publishes CHCs and sites by state obtained from Health Resources and Services Administration’s Uniform Data System. We downloaded number of sites for each state for 2018.

From the Census Bureau’s American Community Survey (ACS), Kaiser Family Foundation calculates each states’ number of non-elderly adults with incomes below 100% of the Federal Poverty Line (FPL), and we downloaded this number. We then divided each states’ number of HPSA designations and FQHC delivery sites by its number of non-elderly poor adults.

#### State’s Health Professional Shortage Areas per Non-elderly Poor Resident

Health Professional Shortage Areas (HPSAs) are (1) Medically Underserved Areas: areas which federal officials determine have health manpower shortages such that healthcare professionals are not accessible; (2) Medically Underserved Populations: population groups which experiences such a shortage; and (3) Medically Underserved Facilities: public or non-profit private medical facilities or other public facilities which experience such a shortage. Working with partners in the states, the federal Health Resources and Services Administration (HRSA) assigns shortage designations to qualifying areas, populations, and facilities which become eligible to receive certain federal resources. All Federally Qualified Health Centers benefit accordingly [42].

We downloaded each state’s number of HSRAs as reported on HRSA website [42]. We then divided each states’ number of HPSA designations by its US census-bureau determined [43] number of non-elderly poor adults.

### Principle Independent Variables

Racial bias was assessed using data from Project Implicit [44], a database of racial bias test results collected over the internet from all states since 2002. We aggregated data from *n* = 1,084,533 non-Hispanic White respondents who completed the implicit and explicit bias assessment from 2013 to 2018 and provided data on their state-of-residence. Following prior work [35, 39], we restricted bias assessments to Project Implicit tests taken by non-Hispanic Whites.

#### Implicit Racial Bias

The Implicit Association Test (IAT) measures implicit (i.e., unconscious, automatic) racial bias by prompting respondents to simultaneously match “African American” or “European American” faces with words indicating positive (e.g., beautiful) or negative (e.g., terrible) qualities. Faster reaction times when matching positive descriptors with White faces and negative descriptors with Black faces indicate stronger Black-negative and White-positive associations. Implicit bias scores range from − 2 to + 2, with negative values representing a pro-Black/anti-White bias, positive values representing an anti-pro-White/anti-Black bias, and 0 representing a neutral score. Respondents who made errors on < 30% of trials or had reaction times < 300 ms on < 10% of trials were excluded, following prior work [31, 45].

#### Explicit Racial Bias

To assess explicit bias, respondents were asked to rank their warmth vs. coldness toward Black people and White people on an 11-point Likert scale ranging from 0 “extremely cold” to 10 “extremely warm.” Following previous work [22], we calculated the difference between the White and Black scores to measure explicit racial bias. Scores range from − 10 to + 10 with negative values representing a pro-Black/anti-White bias, positive values representing an anti-pro-White/anti-Black bias, and 0 representing a neutral score.

### Covariates

We adjusted for potential confounding of the association between Whites’ racial bias and availability of FQHC sites and HRSA designations. We considered states’ African American population proportion as a proxy for bias when selecting covariates because large African American populations are thought to elicit Whites’ bias [10], and because African American populations’ expected association with implicit and explicit bias has been borne out in research [18].

#### Fair/Poor Health

African Americans are overrepresented in potentially more biased states which have less healthy populations [36, 46, 47]. These states should qualify for HPSA designations more than others and they require healthcare resources such as FQHCs. We measured states’ population health status by aggregating responses on The Behavioral Risk Factor Surveillance System, an ongoing, state-based, random-digit-dialed telephone survey of adults aged 18 years and older. Respondents are asked a question strongly correlated with greater healthcare utilization and mortality [48]: whether “in general” their health is excellent; very good; good; fair; or poor. We downloaded each state’s 2018 proportion of non-elderly poor person who rate themselves in fair or poor health.

#### Conservatism

We controlled for residents’ political conservatism to account for states’ small-government, personal responsibility philosophies operating apart from bias. Conservatism is associated with larger African American populations and potentially with bias, and with opposition to safety net programs [10]. In Gallup’s 2018 tracking poll, respondents were asked to describe their political views as “liberal,” “moderate,” or “conservative.” We entered each states’ percent of people rating their views as conservative.

#### Median Household Income

African Americans are overrepresented in poor states [49] which have fewer resources to supplement federal spending on FQHCs and HPSAs and other safety net programs. We therefore controlled for the median household income dollar values for each state. From the American Community Survey, the US Census Bureau calculates and reports each state’s 2018 median household income [50]. We accessed and entered median household income dollar values for each state.

#### Acceptance or Rejection of Medicaid Expansion

Medicaid expansion, which African Americans were denied disproportionately [17], expresses state officials’ willingness to support healthcare access as a matter of public responsibility [51]. Medicaid expansion also spurred FQHC growth by providing 11 billion dollars [25] to double FQHC capacity [37] and bringing an almost 50% increase in service markets served by FQHCs [52]. Medicaid expansion also increased FQHCs’ capacity for Medicaid outreach and enrollment [24].

To adjust for official opposition to the ACA and “government healthcare” [9, 51] as well as adjusting for the role of Medicaid growth in FQHC growth—and to isolate FQHCs and HPSAs as targets of bias from wider ACA disapproval—we controlled for states’ adoption or rejection in 2014 of the ACA’s Medicaid expansion. In a report to Congress, the Medicaid and CHIP Payment and Access Commission [53] listed states that did and did not expand Medicaid when expansion began in 2014. We coded states: accepting = 1, rejecting = 0.

### Analysis

First, we correlated all study variables by computing indicators of states’ rank order agreement. The resulting focus on relative standing eliminates the influence of distributional properties and comports with an established policy focus on comparing states on the basis of higher vs. lower ranking (e.g. [54]).

To test our hypotheses that states with more racial bias would have fewer FQHCs and HPSAs, we regressed each state’s FQHCs per-poor non-elderly adults separately on explicit and implicit bias, along with covariates. We regressed state’s HPSAs per-poor non-elderly adults on the above variables along with FQHCs per-poor non-elderly adult to control for FQHCs also being HPSAs. We transformed FQHC sites and HPSAs per-poor non-elderly adults by taking logarithms to minimize distribution irregularities and, from caution over possible heteroskedasticity, we employed robust standard errors.

## Results

Table 1 presents descriptive statistics. The number of non-Hispanic White tests aggregated per state from 2013 to 2018 ranged from 1118 to 94,410 with mean = 21,266 and standard deviation (SD) = 20,285. The average of states’ racial bias scores was 0.36 (SD = 0.03) for implicit bias and 0.40 (SD = 0.15) for explicit bias, and states averaged 67.6 per 100,000 FQHC sites per non-elderly poor adult and 94.9 per 100,000 for HPSAs. The average of states’ percentages of persons identifying as politically conservative was 36%. States’ average median household income was about $61,500, and states’ average rate of self-reported fair/poor health was 18%. Thirty-one states, or 62%, expanded Medicaid in 2014.

**Table 1:** Descriptive statistics: state’s implicit and explicit bias, conservatism, income, health, ACA Medicaid expansion

	Mean	SD
Dependent variable		
FQHC sites per-poor non-elderly adult	.000676	.000638
HPSAs per-poor non-elderly adult	.000949	.001671
Independent variables		
Implicit bias 2013–2018	.364	.025
Explicit bias 2013–2018	.396	.145
Conservatism (%)	36.08	6.46
Median household income	61,549	10,184
Fair/poor health (%)	18.10	3.14
Expanded Medicaid in 2014 (%)	62.00	.490

Preliminary analysis of rank order correlations (Table 2) revealed the following. FQHCs correlated negatively with explicit bias (rho =  − 0.37, *p* < 0.01) and implicit bias (rho =  − 0.33, *p* < 0.05), and HPSAs correlated negatively with explicit bias (rho =  − 0.51, *p* < 0.01). Implicit and explicit bias themselves proved highly and positively correlated (rho = 0.76, *p* < 0.01). Both FQHCs (rho =  − 0.26, *p* < 10) and HPSAs (rho =  − 0.27, *p* < 0.10) correlated negatively with fair/poor health as trends. FQHCs correlated with accepting Medicaid expansion (rho = 0.28, *p* < 0.05) and HPSAs correlated positively with conservatism (rho = 0.36, *p* < 0.05).

**Table 2:** Rank order correlations: state’s implicit and explicit bias, conservatism, income, health, ACA Medicaid expansion

	Explicit bias	Implicit bias	Conserva-tism	Median income	Fair/poor health	Expanded Medicaid
FQHCs^1^	− .37**	− .34*	− .09	− .02	− .27^a^	.28*
HPSAs^2^	− .51**	− .07	.36*	− .20	− .26^a^	.07
Explicit bias		.76**	.36**	− .31*	.11	− .23
Implicit bias			.10	− .20	.27^a^	− .11
Conservatism				− .73**	.43**	− .59**
Median income					− .69**	.36**
Fair/poor health						− .15

^1^*FQHC*, sites as proportion of state’s non-elderly population < 100% Federal Poverty Line

^2^*HPSA*, Health Professional Shortage Area designations state’s non-elderly population < 100% Federal Poverty Line

*n* = 50; ^a^*p* < .10, *p < .05, **p < .01

Regression results from testing study hypotheses for FQHCs after covariate adjustment (Tables 3 and 4) revealed that implicit racial bias was inversely associated with FQHCs (*b* =  − 3.11, SE = 1.41, *p* < 0.05). Explicit racial bias too was inversely associated with FQHCs (*b* =  − 0.78, SE = 0.29, *p* < 0.05). Along with implicit and explicit bias, accepting Medicaid expansion was associated as a trend in the model which included implicit bias (*b* = 0.24, SE = 0.02, *p* < 0.10), and associated statistically significantly in the model which included explicit bias (*b* = 0.23, SE = 0.11, *p* < 0.05).

**Table 3:** Log of FQHC availability regressed on state’s implicit bias, state’s implicit bias, conservatism, income, health, and ACA Medicaid expansion

	*β*	Robust SE	95% CI
Intercept	0.88	1.44	− 2.03 3.78
Implicit bias	− 3.11*	1.41	− 5.95 − 0.28
Conservatism	0.01	0.02	− 0.02 0.04
Median income	− 0.00	0.00	− 0.00 0.00
Fair/poor health	− 0.02	0.02	− 0.06 0.02
Expanded Medicaid	0.24^a^	0.02	− 0.03 0.51

*R*^2^ = .19, *p* <.05. ^a^*p* < .10, *p < .05, **p < .01

**Table 4:** Log of FQHC availability regressed on state’s explicit bias, conservatism, income, health, and ACA Medicaid expansion

	*β*	Robust SE	95% CI
Intercept	0.04	1.36	− 2.69 2.78
Explicit bias	− 0.78**	0.29	− 1.36 − 0.21
Conservatism	0.18	0.02	− 0.02 0.05
Median income	− 0.00	0.00	− 0.00 0.00
Fair/poor health	− 0.03	0.02	− 0.07 0.01
Expands Medicaid	0.23*	0.11	0.01 0.45

*R*^2^ = .24, *p* < .01. *p < .05, **p < .01

For HPSAs (Tables 5 and 6), covariate-adjusted regression results from testing study hypotheses revealed that implicit racial bias was inversely associated (*b* =  − 10.87, SE = 2.55, *p* < 0.01). Explicit racial bias too was inversely associated with HPSAs (*b* =  − 1.29, SE = 0.61, *p* < 0.05). Several covariates proved significantly associated with HPSAs. These were conservatism (implicit bias: *b* = 0.08, SE = 0.03, *p* < 0.01; explicit bias: *b* = 0.09, SE = 0.03, *p* < 0.01), fair/poor health (implicit bias: *b* =  − 0.12, SE = 0.03, *p* < 0.01; explicit bias: *b* =  − 0.16, SE = 0.03, *p* < 0.01), and FQHCs (implicit bias: *b* = 0.81, SE = 0.34, *p* < 0.05; explicit bias: *b* = 0.83, SE = 0.34, *p <* 0.01).

**Table 5:** Log of HPSAs regressed on state’s implicit bias, conservatism, income, health, ACA Medicaid expansion, and FQHCs

	*β*	Robust SE	95% CI
Intercept	− 3.12	1.99	− 7.13 0.88
Implicit bias	− 10.87**	2.55	− 16.01 − 5.73
Conservatism	0.08**	0.03	0.02 0.13
Median income	− 0.00	0.00	− 0.00 0.00
Fair/poor health	− 0.12^**^	0.03	− 0.18 − 0.07
Expands Medicaid	0.25	0.19	− 0.13 0.64
Log FQHCs	0.81*	0.34	0.12 1.50

*R*^2^ = .64, *p* < .01. *p < .05, **p < .01

**Table 6:** Log of HPSAs regressed on state’s explicit bias, conservatism, income, health, ACA Medicaid expansion, and FQHCs

	*β*	Robust SE	95% CI
Intercept	− 6.62**	1.84	− 10.32 − 2.91
Explicit bias	− 1.29*	0.61	− 2.25 − 0.06
Conservatism	0.09**	0.03	0.04 0.15
Median income	− 0.00	0.00	− 0.00 0.00
Fair/poor health	− 0.16**	0.03	− 0.21 − 0.10
Expands Medicaid	0.34	0.21	− 0.09 0.77
Log FQHCs	0.83*	0.34	0.15 1.50

*R*^2^ = .58, *p* < .01. *p < .05, **p < .01

Exclusively state-level modeling accounted for significant variation in FQHCs and HPSAs. The models explained 19% and 24% of FQHC variation (Tables 3 and 4) and 64% and 58% of HPSA variation (Tables 5 and 6).

## Discussion

This study found that states where White residents exhibit more explicit and implicit bias toward African Americans have fewer Federally Qualified Health Care delivery sites available for healthcare and fewer designated Health Professional Shortage Areas to identify poor African Americans and other economically disadvantaged citizens with limited healthcare access. The findings suggest that anti-African American bias may deter recognizing healthcare access limitations and making FQHCs available to all vulnerable citizens in need.

Racial bias remains a viable explanation for fewer FQHC sites and fewer designated HPSAs after adjusting for states’ ability to contribute more resources to safety net spending from higher household income levels, from relatively poor health status, and from a small government, self-reliance-preferring conservative philosophy. Conservatism sometimes competes with racial antipathy as theorists and investigators try to explain opposition to safety net programs [10] and other social policies [40], but conservatism did not explain the racial bias-FQHC association in the present study.

Population health also is associated with state African American population representation [46, 47] and state residents’ poor health. Notably, states with *more* citizens reporting fair or poor health had *fewer* HPSA designations, but states with *more* citizens with fair or poor health should have be *more* HPSAs. The finding underscores the importance of state-level factors beyond population health, including cultural, political, and administrative factors, in certifying HPSAs. In any event, bias remained associated with FQHC availability and with identifying more HPSAs even after adjusting for the potential influence of poor health on racial bias.

Why did states’ White bias account for more FQHCs and HPSAs? As with other federal net programs [19], state governments can embrace, remain neutral, or restrict their FQHC and HPSA participation by taking or neglecting actions that would support FQHCs and HPSA designations. Contributing to FQHC patchwork budgets, some states provide direct state funding for FQHC operations and uncompensated care and for service expansion; some provide seed money for center start-up [21]. Some states channel special purpose funds to achieve population heath objectives (e.g., immunization targets), and some states facilitate access to information technology, including electronic medical records and patient registries. States sometimes use state-operated purchasing mechanisms to lower purchase costs of necessary supplies [21]. Furthermore, vigorous state-wide Primary Care Associations (PCAs) can organize local actors to stimulate state-wide coordination of common programming and funding efforts, as well as advocating to state officials and practice associations for FQHC support [21].

Medicaid is a critical source of FQHC financing and, because Medicaid is a state-federal program [53], state Medicaid official make key Medicaid decisions. Along with accepting or rejecting Medicaid expansion and with it greater or lesser Medicaid coverage [55], states can ease Medicaid enrollment procedures and they can seek waivers to reach out to populations with special needs [21]; they can impose work requirements [56], charge Medicaid copayments, and otherwise impose coverage restrictions on Medicaid-financed recipients of care [21]. Medicaid barriers reduce incentives for Medicaid patients to seek care and can increase FQHCs’ proportional burden of uncompensated care.

HPSAs too are subject to the preferences and actions of state officials. States collect and prepare data to secure federal designation of HPSAs through Primary Care Offices (PCOs), usually located in States Health Departments [21]. PCOs obtain and update HPSA designations by collaborating and providing technical assistance to local agencies and communities. Local actors often lack resources for independent participation and they face competing priorities [57], especially in states where FQHCs do not seem economically viable due to limited state supplemental funding and limited state-wide advocacy and organizational support. It is reasonable to believe that, operating in state administrative hierarchies, by subtle and direct means, climates of disapproval can circumscribe PCO efforts to prepare and forward what would otherwise be HPSA designations.

More research is needed to clarify exactly how states influence FQHCs and HPSA designations. Studies should identify a menu of potentially consequential state actions, define them in clear-cut terms, and assess their impact on FQHC applications and success and on HPSA activities leading to HPSA designations. The impact of state actions is important to understand on its own, and as a mediator of unwelcoming racial and sociopolitical environments.

This study’s findings should be interpreted keeping several limitations in mind. The study’s design is cross sectional and non-experimental which precludes causal interpretation of correlations between racial bias and FQHC availability and HRSA designations. Unmeasured correlates might confound the observed associations. One outcome measure, FQHCs per non-elderly, poor adult, was somewhat restricted in variation and study findings, although statistically significant, might have been artificially constrained.

Project Implicit respondents are self-selected. Common reasons for participation include class assignments and racial bias trainings [58]. On average, Project Implicit respondents tend to be younger and comprised of more women than the general population [59]. However, in a 2019 validation paper, Hehman et al. (2019) found high convergent validity when comparing state-level implicit and explicit racial bias data from Project Implicit with racially charged Google searches and nationally representative racial attitude data from the Pew Research Center. Others have shown robust associations between aggregate racial bias data from Project Implicit and a range of important health and social inequities, including COVID-19 mortality rates [34], chronic disease [35, 60], adverse birth outcomes [33], and racial inequities in income [39, 61], police killings [62], and school discipline practices [63]. Thus, the Project Implicit data likely provide a valid measure of area-level racial attitudes, despite self-selection of the sample.

Despite these limitations, findings in the present study convincingly suggest that state-levels of anti-African American bias are inversely associated with the presence of FQHCs, a leading safety net healthcare provider and critical resource for the advancement of racial and socioeconomic health equity. The results demonstrate that widely shared biased personal views of residents of a state can underwrite anti-safety net programming decisions. The resulting institutionalized bias denies healthcare resources to African Americans and to other poor residents of higher bias states.

## Conclusion

Operating in the realm of healthcare, Whites’ anti-Black bias can contribute to the limiting of FQHC availability and HPSA designations to the detriment of poor people who disproportionately are African American. Vulnerable people are denied recognition of limited healthcare access from HPSA designation and opportunities for preventive care and treatment by FQHCs. Greater understanding is needed of exactly how bias might contribute to bringing about these barriers in order to and improve the health of African American and other poor citizens and, by this and other means, to increase healthcare equity.
